# Mobility assessment in people with Alzheimer disease using smartphone sensors

**DOI:** 10.1186/s12984-019-0576-y

**Published:** 2019-08-14

**Authors:** Pilar Serra-Añó, José Francisco Pedrero-Sánchez, Juan Hurtado-Abellán, Marta Inglés, Gemma Victoria Espí-López, Juan López-Pascual

**Affiliations:** 1 UBIC, Departament de Fisioteràpia de la Universitat de València, C/ Gascó Oliag, 5, 46010 Valencia, Spain; 2 Instituto de Biomecánica de Valencia, Universidad Politécnica de Valencia, Edificio 9C. Camino de Vera, s/n, 46022 Valencia, Spain

**Keywords:** Functional mobility, Alzheimer’s disease, Android device, Dual-task, Gait

## Abstract

**Background:**

Understanding the functional status of people with Alzheimer Disease (AD), both in a single (ST) and cognitive dual task (DT) activities is essential for identifying signs of early-stage neurodegeneration. This study aims to compare the performance quality of several tasks using sensors embedded in an Android device, among people at different stages of Alzheimer and people without dementia. The secondary aim is to analyze the effect of cognitive task performance on mobility tasks.

**Methods:**

This is a cross-sectional study including 22 participants in the control group (CG), 18 in the group with mild AD and 22 in the group with moderate AD. They performed two mobility tests*,* under ST and DT conditions, which were registered using an Android device. Postural control was measured by medial-lateral and anterior-posterior displacements of the COM (*MLDisp* and *APDisp*, respectively) and gait, with the vertical and medial-lateral range of the COM (*Vrange* and *MLrange*). Further, the sit-to-stand (*PStand*) and turning and sit power (*PTurnSit*), the total time required to complete the test and the reaction time were measured.

**Results:**

There were no differences between the two AD stages either for ST or DT in any of the variables (*p* > 0.05). Nevertheless, people at both stages showed significantly lower values of *PStand* and *PTurnSit* and larger *Total time* and *Reaction time* compared to CG (*p* < 0.05). Further, *Vrange* is also lower in CDR1G than in CG (*p* < 0.05). The DT had a significant deleterious effect on *MLDisp* in all groups (*p* < 0.05) and on *APDisp* only in moderate AD for DT.

**Conclusions:**

Our findings indicate that AD patients present impairments in some key functional abilities, such as gait, turning and sitting, sit to stand, and reaction time, both in mild and moderate AD. Nevertheless, an exclusively cognitive task only influences the postural control in people with AD.

## Introduction

Alzheimer’s disease (AD) is the primary cause of irreversible dementia among elderly people [1]. The clinical hallmark of early AD is episodic memory impairment, which is accompanied by changes in executive control, predominantly inhibitory control [2, 3].

This executive control deficit and the hyperexcitability of the motor cortex affect gait [3, 4]. Accordingly, several studies have suggested that changes in gait might precede AD diagnostic [6, 7]. Overall, people with AD present lower gait speed [8], shorter stride length [8, 9] and greater stride time variability [10] than their healthy counterparts. Further, these alterations in the kinematic parameters of gait have been previously associated with an increased risk of falls in this population [11].

This implies the necessity of assessing functional tasks such as gait and other more complex daily life activities (DLA) that require neuromuscular coordination planning (i.e. sitting down and getting up from a chair or turning around) in this population. This mobility function monitoring could help to predict the physical progression of the disease, since these tasks require from the integrative function, both cognitive and behavioral components, and are the basis of the ability to manage independent DLA [5]. However, these functional activities, in a real context, are not usually conducted alone but are performed simultaneously with other activities whose execution also require attention; this is known as dual tasking. Carrying out different tasks simultaneously, with diligence, requires a constant shift of the attention between the primary task (gait) and the secondary task [12]. Nevertheless, as reported, attention control, specifically the ability to divide attention in this population, is impaired [13], and also it is the prioritization of gait when performing the secondary task [5], so dual-task mobility assessment becomes even more relevant for them.

Establishing clinical markers that could predict functional mobility status in people with AD, both in single-task and dual-task activities, is important to identify subtle signs of early-stage neurodegeneration in order to understand the early neuropathological changes, prevent physical decline and better plan the treatment protocols. It is known that earlier intervention is likely to be more effective and may truncate the ill effects of secondary events due to inflammatory, oxidation, excitotoxicity, and apoptosis [14]. Further, treatment programs including dual-task activities, promoting change in attention’ prioritization, can improve mobility function and therefore reduce the risk of falling [5].

There are several ways to perform functional assessments, however, due to the cognitive condition of this population, auto-reported questionnaires are not the best option. By contrast, objective tests are more appropriate in these patients, preferably short tests because of their attention control impairment [15]. In this regard, some studies have used the *Timed-Up and Go* test in this population because it is simple and quick [16] and includes, besides walking in a straight line, other tasks such as turning or rising from a chair that require more cognitive resources than just walking [17]. Nevertheless, in general, the resultant variable of this test is the time to complete it [9], even when a modified version of TUG is used [17].

To obtain more information, not only about the speed but also about the quality of movement in this population, other studies have conducted the assessments using likewise objective, yet more sophisticated mechanisms, such as video cameras [8, 9] or pressure sensor devices [6, 18]. Nevertheless, this approach, although necessary to assess the functionality of these patients, requires the use of expensive tools, high-level training of the clinical personnel conducting the assessments and is thus constrained to the laboratory environment.

Based on the above, this study aims to compare the performance quality of several tasks included in a simple mobility test using sensors embedded in an Android device, among people at different stages of Alzheimer and people without dementia. We further analyzed the effect of cognitive task performance on the functionality.

## Methods

### Participants

The study design was cross-sectional including two groups. The CG was formed by 22 age-matched participants without dementia and the Alzheimer group (AG) included 40 participants, diagnosed with AD by a specialist physician using the revised NINCDS-ADRDA criteria [14]. Only people at stages 1 and 2, according to the Clinical Dementia Rating (CDR) [19], were included. Since our purpose was to detect subtle mobility impairments in the earliest stages of the disease, we used this classification that provides information about the social participation, domestic chores and personal care and selected only the first two classifications (i.e. mild and moderate). Therefore, the AG was split into two groups, CDR1G (*n* = 18) and CDR2G (*n* = 22), respectively. The sample size was determined by the resultant effect size of the variable “Time” in a previous study [20]. We set the type I error at 5% and a statistical power of 80%.

A purposive sampling that lasted 5 months was used to select the participants. The AG recruitment was conducted from Alzheimer associations whilst the participants in the CG were recruited through advertisements at leisure facilities for elderly people.

Inclusion criteria, for both groups, were the ability to walk at least 10 m without walking aids and the availability to participate in the assessments. Exclusion criteria, also for both groups, were the presence of motor alterations after stroke, neurological disorders that interfered in mobility and severe uncorrected visual or auditory disorders.

Furthermore, to ensure homogeneity between groups, their levels of anxiety and depression were measured with the *Hospital Anxiety and Depression scale* [21] and their fear of falling, with the *Fear of falling questionnaire* [22].

### Mobility assessment with android device

**Fig. 1:**
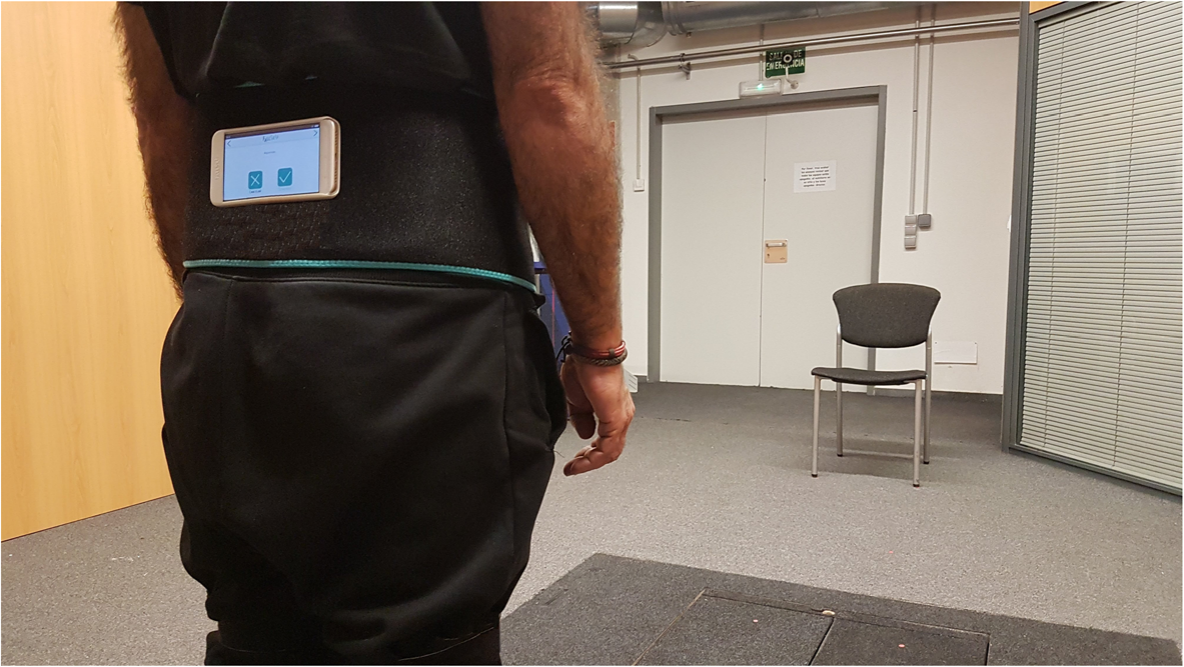
Participants’ instrumentation

Mobility assessment was performed using the system FallSkip® (Biomechanical Institute of Valencia) which is a software running on an Android Device (Xiaomi Redmi 4x Model MAG138). FallSkip® assisted the evaluator throughout the test by means of visual indications on the screen and acoustic tones that fostered the correct performance of the protocol. The data were acquired via the device sensor, specifically, High-Performance 6-Axis MEMS MotionTracking™ composed of 3-axis gyroscope (gyro), 3-axis accelerometer (acc), and a Digital Motion Processor™ (TDK- ICM-20689) at 100 Hz. A custom specific software was developed in Python to calculate the variables from the sensor raw data. First of all, the height and weight of the participants were measured and subsequently used to calculate the clinical dependent variables. The device was then horizontally attached with an elastic belt just below the posterior superior iliac crests (Fig. 1).

**Fig. 2:**
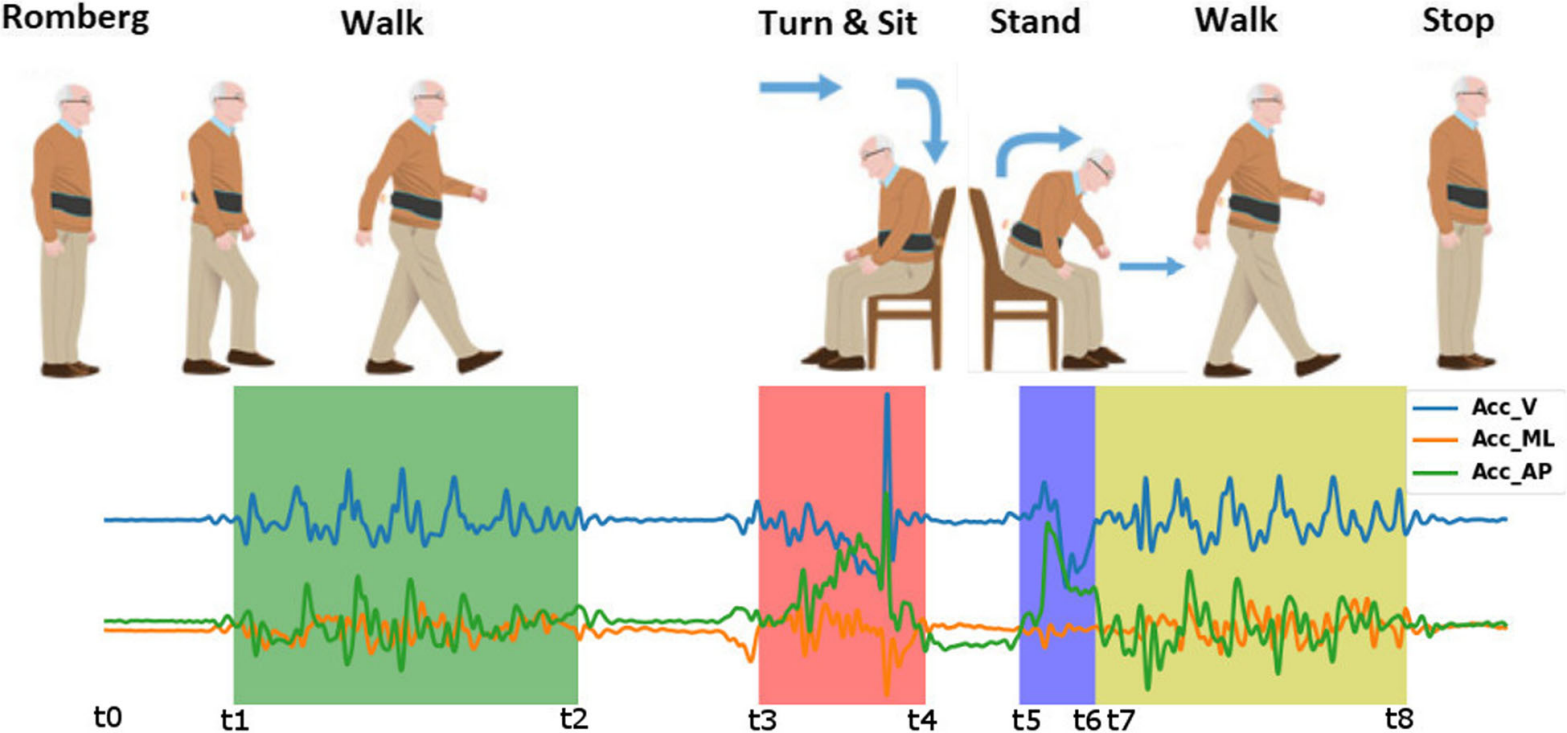
Delimitation of tasks performed during the test. The three lines below the figures represent the accelerometer signal in the different tasks of the test. Green shade: walking; Red shade: turning and sitting; Blue shade: Getting up from the chair; Yellow shade: walking back to the starting position; Acc_V: accelerometer signal in the vertical axis; Acc_ML: accelerometer signal in the medial-lateral axis; Acc_AP: accelerometer signal in the anterior-posterior axis

The assessment procedure consisted of performing a mobility test with postural control, locomotion and sitting to standing components (Fig. 2). Firstly, the participants should remain in a standing position with the arms alongside the body for 30s. After that, an acoustic signal sounded and immediately, the participant should start walking a 3-m stretch as fast as safely possible. When the distance was covered, they should stop for 3 s and then turn around and sit down on a chair. They should remain seated for 3 s before getting up and returning to the starting position. Reliability for this assessment protocol was previously established by our group [23].

This test was conducted twice, once as a single task (ST), and a second time additionally including a cognitive task that consisted in telling a real story of their own choice (DT). In this dual-task test, no instructions were given regarding focusing on the task (motor or cognitive). The rater stood behind the participants during the test to prevent falls during the assessments. Furthermore, several trials were conducted before the test started to allow the participants to familiarize themselves with the tests.

The participants were instructed to wear comfortable clothing and their usual walking shoes, to avoid vigorous exercise the day before the tests, and to bring any necessary visual or auditory aids before the assessment.

### Android data analysis and outcomes

All sensor raw data were processed according to the following procedures [24, 25]: (i) linear interpolation to overcome the non-constant sample rate of the Android device; (ii) signal filtering with a low-pass Butterworth filter (fourth-order zero-lag at 20 Hz).

Time events were manually identified to split up the recorded data in the tasks under interest (Fig. 2). The identification was conducted by one evaluator and confirmed by a second one, according to the following criteria:

- t0: End of the postural control test at 30 s after the test started
- t1: Beginning of the walking when acc signals grow in activity
- t2: Walking stop when acc signals stabilize close to baseline (t0) values, before the 3 s pause
- t3: Beginning of the turning with a steady change of acc and/or gyro magnitudes
- t4: End of the sitting down when acc and gyro signals stabilize, prior to the 3 s pause
- t5: Beginning of the standing up with a steady change of acc and gyro magnitudes
- t6: End of the standing up when acc and gyro magnitudes return to baseline (t0) values
- t7: Beginning of going back at t6 or when acc signals grow in activity after a pause
- t8: End of the walking back when acc signals stabilize close to baseline (t0) values

Based on the raw data from the sensors, some postural control, gait and functionality variables were calculated.

Two variables were calculated for the postural control variables: i. M*edial-lateral displacement (MLDisp)*: 90th percentile of the ML excursion of the center of mass (COM), measured in mm and calculated by double integration of the acc signal [26] and an inverted pendulum model [25] ii. A*nterior-posterior displacement (APDisp):* 90th percentile of the AP excursion of the COM, measured and calculated in the same way as above. Both are common variables used in the assessment of the postural steadiness as refer to the COM displacement [27].

With respect to gait analysis, two variables were measured: i. *Vertical range (Vrange)*: vertical movement of the COM, measured in mm, taking the average of going back (t7,t8) and forth (t1,t2) the 3-m distance, and ii. *Medial-lateral range (MLrange)*: horizontal movement of the COM, measured in mm, taking the average of going back and forth the 3-m distance. Both were calculated by double integration of the acc signal [28]. Vrange is a measurement of the energy cost [28–30] while MLrange, beside the energy cost, refers to the dynamic stability during gait and supposes a useful variable to measure the impact of a secondary task during gait [31, 32].

Likewise, turning around and sitting down and getting up from a chair were also monitored and two variables were calculated: i. *Turn-to-sit power* (*PTurnSit*): estimated mean power, measured in watts, generated in turning around and sitting in a chair (t3,t4); and ii. *Sit-to-stand power* (*PStand*): estimated mean power, measured in watts, generated in getting up from the chair (t5,t6). Both variables were estimated by the trajectory of the COM and the weight and height of the participant during the movements [33]. These are complex motor DLA that require cognitive planning and coordination of the neuromuscular systems to regulate the displacement of the COM [34]. The variables computed provide clinical information beyond the time to complete the task.

Finally, two variables related to time, measured in seconds, were calculated: i. *Total time (Time)*: the sum of time needed to complete all the tasks: 2 phases of gait (t2-t1), (t8-t7), sit (t4-t3) and stand (t6-t5) and ii. *Reaction time*: time passed from the acoustic signal to gait initiation. Speed is the most common variable used when describing gait [7] and reaction time has been proved useful in predicting cognitive performance [35].

### Statistics

Statistical analysis was performed using SPSS software Version 24 (SPSS Inc., Chicago, IL, USA). Standard statistical methods were used to obtain the mean as a measure of central tendency and the standard deviation (SD) as a measure of dispersion. For the inferential analysis, a mixed-model multivariate analysis of variance (MANOVA) was performed to establish the effects of the between-subjects factor ‘group’ with three categories (CDR1G, CDR2G and CG) and the within-subjects factor ‘condition’ with two categories (single-task and dual-task) on the dependent variables (i.e. *MLDisp, APDisp, Vrange, MLrange. PTurnSit, PStand, Time and Reaction Time*). The results showed the effect of the factors’ interaction and also of the single factors (i.e. ‘group’ main effect and ‘conditions’ main effect) Pairwise comparisons were performed with the Bonferroni correction. A *p*-value of 0.05 was accepted as the level of significance.

## Results

### Participants

The AG included 40 individuals with a mean (SD) age of 78.58 (6.34) years. When the group was split into two subgroups depending on the CDR classification, CDR1G was 76.78 (6.73) years and CDR2G, 80.05 (5.74) years. The CG, with 22 people, showed a mean (SD) age of 75.5 (5.61) years. Table 1 shows that there were no significant differences in any of the anthropometric or clinical variables among groups (*p* > 0.05).

**Table 1:** Clinical and anthropometrical profile of the participants stratified by groups

	CDR1G	CDR2G	CG	*P* value
Weight (kg)	73.91 (14.08)	70.02 (11.09)	73.26 (11.59)	0.56
Height (m)	1.54 (0.11)	1.54 (0.07)	1.59 (0.07)	0.14
Anxiety	5.83 (3.28)	5.00 (3.53)	6.94 (5.35)	0.34
Depression	5.67 (3.25)	4.86 (3.04)	5.72 (3.53)	0.64
FFQ	44.67 (3.4)	44.86 (8.4)	45.67 (5.79)	0.88

The data are expressed as mean (SD)

*FFQ* Fear of falling questionnaire, *CDR1G* Group of people with Alzheimer classified as stage 1 by the Clinical Dementia Rating, *CDRG* Group of people with Alzheimer classified as stage 2 by the Clinical Dementia Rating, *CG* Control group

### Mobility assessment

No significant multivariate and univariate interactions between groups and condition were obtained (*p* > 0.05). Table 2 shows the univariate main significant effect of each factor isolated (i.e. group and conditions) and the pairwise comparisons between groups for each condition and between conditions in each group.

**Table 2:** Descriptive and comparison of the mobility variables between groups and conditions

		CDR1G	CDR2G	CG	Group main effect		Conditions main effect	
					*F* (1,59)	*p*	*F* (1,59)	*p*
MLDisp (mm)	ST test	7.76 (4.29)	7.39 (3.36)	8.34 (4.91)	0.27	0.766	23.54	**< 0.001**
	DT test	12.01 (6.47)^a^	14.62 (8.96)^a^	13.46 (10.09)^a^				
APDisp (mm)	ST test	23.76 (19.55)	24.78 (10.89)	20.62 (10.58)	4.01	**0.023**	8.62	**0.005**
	DT test	27.81 (16.66)	39.59 (21.15)^ab^	23.46 (11.01)				
MLrange (mm)	ST test	57.53 (21.97)	56.37 (19.8)	51.59 (17.75)	1.20	0.307	4.66	**0.035**
	DT test	66.66 (28.5)^a^	57.96 (21.98)	52.75 (18.31)				
Vrange (mm)	ST test	16.23 (5.87)^b^	17.19 (6.31)	20.97 (5.17)	2.16	0.124	0.47	0.493
	DT test	16.65 (6.18)	17.77 (6.8)	18.88 (4.75)^a^				
PStand (W)	ST test	159.39 (51.22)^b^	155.41 (40.75)^b^	195.43 (43.6)	5.46	**0.007**	0.00	0.972
	DT test	161.26 (58.14)	155.94 (35.58)^b^	192.37 (57.84)				
PTurnSit (W)	ST test	55.56 (22.23)^b^	48.19 (11.91)^b^	76.58 (19.37)	18.75	**< 0.001**	1.07	0.305
	DT test	50.68 (18.09)^b^	51 (18.53)^b^	87.53 (35)^a^				
Time (s)	ST test	21.18 (4.27)^b^	21.78 (3.92)^b^	15.99 (2.53)	17.02	**< 0.001**	3.67	0.060
	DT test	22.41 (3.35)^b^	22.01 (4.83)^b^	16.91 (3.77)				
Reaction time (s)	ST test	1.89 (0.83)^b^	1.73 (0.74)^b^	1.07 (0.24)	1.89	0.161	5.39	**0.024**
	DT test	1.93 (0.73)	1.93 (0.9)	1.89 (1.7)^a^				

Data are expressed as mean (SD)

*CDR1G* Group of people with Alzheimer classified as stage 1 by the Clinical Dementia Rating, *CDRG* Group of people with Alzheimer classified as stage 2 by the Clinical Dementia Rating, *ST* Single task, *DT* Dual task, *MLDisp* Medial-lateral displacement, *APDisp* Anterior-posterior displacement, *MLrange* Medial-lateral range, *Vrange* Vertical range, *PStand* Sit-to-stand power, *PTurnSit* Turn-to-sit power

^a^significant differences with the Single-task test

^b^significant differences with the control group (CG)

Bold type indicates a significant main effect of group or conditions (*p* < 0.05)

There were no differences between the two stages of Alzheimer disease in either ST or DT in any of the variables (*p* > 0.05). Nevertheless, people with AD (both stages) showed significantly lower values of *PStand* and *PTurnSit* and higher values of Total time and reaction time compared with CG (*p* < 0.05); this last variable only under the ST condition. Further, CDR1G present larger ML range in ST than CG and CDR2G showed larger *APDisp* in DT than CG (*p* < 0.05).

The effect of the condition was significant in all groups only for *MLDisp,* which showed larger values in DT than in ST. Moreover, the *APDisp* in DT was larger than that obtained in ST only in the CDR2G. Only the CG showed significant differences between conditions in the *Vrange* and *PTurnSit*.

## Discussion

This study defines mobility alterations in people with Alzheimer’s disease, at different stages of evolution, by means of an easy-to-use Android mobile phone, not only considering the time needed to achieve certain functional tasks but also evaluating the performance of these tasks under different conditions. The assessment consisted of the analysis of several daily life activities in a single test using a single device (Smartphone). This is a novelty, since, on the one hand, previous studies have usually focused on the analysis of gait or balance in an isolated way [36, 37], and on the other, measurement procedures have required the use of two or more sensors [38, 39].

In general, the results show that this novel form of mobility assessment allows differentiating the functional capacities between people without dementia and people with Alzheimer’s disease, for either stage. However, there are no significant differences between the two stages of the disease in any of the variables analyzed. This is consistent with the results of previous studies [9, 40, 41] some of which use even more sophisticated tools such as video-photogrammetry with seven cameras [42] or electronic pressure walkway [18] with the exception of Nakamura et al. whose participants classified as moderate AD (CDR2) showed lower gait speed than those with mild AD [43].

Of the postural control variables analyzed, only *APDisp* during the dual task, showed significant differences, being greater in participants with moderate AD than in the preserved cognition group. On the contrary, *MLDisp* showed no significant differences between groups. This may be related to the fact that body stability in the anterior-posterior direction is managed by a functional active ankle, hip and trunk strategy that makes the movement of the COM possible. However, the range of motion of the joints involved in the plane of medial-lateral stability is very small, so an impairment may be more related to anatomical than to functional mechanisms. Therefore, failure in the balance controlling mechanisms of the central nervous system would more readily affect the anterior-posterior balance [42]. Furthermore, this result is only obtained in DT when the attention is focused on the secondary task and the postural control is performed mostly automatically. In line with these results, the medial-lateral displacement of the COM while walking (*MLrange*), which provides supplementary information about the medial-lateral stability and metabolic cost during gait [31], did not differ between groups.

When the walking task was analyzed, the results showed that the vertical range of the COM during gait (*Vrange*) presented differences between groups during the ST, being 29.21% higher in CG than in CDR1G and the values from the CG being 23.29% higher than those obtained by the CDRG2, although the latter comparison did not achieve the level of significance. The vertical range of the COM has previously been associated with metabolic cost during gait [44]. A lower vertical range is associated with greater energy expenditure because of greater mechanical work performed at the hip, knee, and ankle joints [29]. Based on the inverted pendulum theory [39], whereby the stance leg acts as an inverted pendulum during gait, the exchange between potential and kinetic energy during each stride requires a certain amount of vertical lift of the CoM. Therefore, the vertical displacement (*Vrange)*, which in our study is reduced in the Alzheimer groups, may relate to inefficient gait.

Since not only postural control and gait are indicators of the risk of falls or functional mobility, other functional tests were analyzed. The sit-to-stand task is an essential activity in daily life and requires the coordination of the neuromuscular systems to regulate the displacement of the COM and to control postural alignment. Indeed, the sit-to-stand activity has been included in therapeutic programs in people with dementia because it can help to slow the decline in mobility and function in activities of daily living in this population [45]. Our results showed that the power generated to stand up from the chair is greater in people without dementia than in AD patients, at either stage, during ST and only greater than in moderate AD patients during the DT test. An efficient STS task requires an appropriate amount of energy to accelerate the center of mass from the sitting position to standing position [46]. To the best of our knowledge, there are no previous studies assessing sitting and standing from a chair using Smartphones. However, in this vein, a previous study using video-photogrammetry demonstrated that people with Alzheimer’s reduce the horizontal motion of the trunk and thigh motions during the forward displacement and before the upward movement. This altered kinematic pattern of movement, besides increasing the gravity torque around the knee joint, slows the motion and requires greater muscular force to perform the same movement [34], this being consistent with the reduction in power. This suggests that AD subjects may have lost their ability to prepare and execute efficient body movements, probably because of the impairment to integrate the higher levels of motor process and the dynamics of the external environment [34].

The other task assessed in this study was sitting, that also included turning around because turning involves more inter-limb coordination and modification of locomotor patterns and requires frontal lobe cognitive executive function and attention [40]. Both AD groups in our study showed lower power than the CG (in both tasks), which can be associated with the impaired attention control and is in line with the results obtained in the sit-to-stand task.

With regard to the time variables, our results showed that more time was required to complete the test in both AD groups compared to the CG for both conditions. This is consistent with previous studies that have reported a reduced walking speed [6, 8] and increased time to perform a similar test (i.e. TUG) [16], which in turn are associated with adverse events, including falls in healthy older adults and in AD population [41]. Nevertheless, we did not achieve significant differences in speed between AD groups in contrast with reported by Nakamura et al. [43]. This discrepancy might be related to the instructions given to the participants. While in their study they used the participants comfortable walking pace, we instructed them to walk as fast as safely possible. In our study, the two stages of AD analyzed in our study are not enough different to observe differences when they are asked to conduct an energy-demanding task, like walking and performing the mobility tasks as fast as safely possible. Furthermore, our study showed that the time lapse between the start signal and gait initiation (*Reaction time*) was longer in people with AD than in their non-dementia counterparts suggesting that the reaction time is impaired in people with AD. This is in consonance with previous literature that also showed a larger reaction time in people with dementia [47]. Although it would have been of interest to analyze variability in reaction time, as a measurement of neural integrity [48], using more than one repetition, *Reaction time* allows us, with this simple test, to determine differences between people with AD and people without cognitive impairment and therefore include this topic in therapeutic programs.

Although we pretend to explore the mobility task impairment in the early stages of AD, one limitation of our study has been, precisely, to restrict the participants’ recruitment to two those stages. A study including more advanced stages of the disease or even a first stage before the AD diagnosis (mild cognitive impairment) could better enlighten the progression of mobility impairment.

Regarding the secondary aim, the effect of dual-task performance in the participants was significant only for the postural control variables, the values of *MLDisp* being larger in all the groups and the value of *APDisp* only in the moderate AD group. These variables are derived from the task of standing during 30s in which no volitional movements are performed. Thus, the attention control impairment present in this disease may jeopardize the postural control ability when the patient focuses on recalling a story. The other the variables, in which the participant should move voluntarily, were not influenced by DT in the AD participants. Only the CG was influenced by DT, specifically *PTurnSit*, which was larger in the DT, and *Vrange*, which was lower in DT. The absence of poorer results in DT in people with AD could be due to the fact that these tasks require attention control and cognitive resources that is also compromised when single-tasking. Therefore, their results could not become significantly more impaired during dual-task. Nevertheless, this is in conflict with the results obtained by Ansai et al. who reported a significantly longer time in people with AD in dual-task performance.

However, our dual task consisted only of telling a real story while performing the test, while these authors used a mixed cognitive and motor type dual task, in which the gestures necessarily affected the execution of the test. Our study intended to use an exclusively cognitive and real-life task without being dependent on the training level because it has already been shown that more complex dual tasks are not suitable for this type of population [17]. Perhaps more complex cognitive tasks could be used in the future, without needing a high intellectual level, in order to identify functional components that differentiate AD stages.

## Conclusions

The proposed functional assessment method shows that people with AD present impaired functional abilities, such as gait, turning and sitting, sitting to standing and reaction time. Nevertheless, an exclusively cognitive task only affects the postural control in people with AD. Our findings support that the use of an Android device is a feasible and simple way of assessment in this population.

## Data Availability

The datasets used and/or analyzed during the current study are available from the corresponding author on reasonable request.

## Abbreviations

AD: Alzheimer disease
AG: Alzheimer group
APDisp: Anterior-posterior displacements of the COM
CDR: Clinical Dementia Rating
CDR1G: Group composed of people whose symptoms belong to the stage 1 of the Clinical Dementia Rating
CDR2G: Group composed of people whose symptoms belong to the stage 2 of the Clinical Dementia Rating
CG: Control group
DT: Dual task
FFQ: Fear of falling questionnaire
MANOVA: Multivariate analysis of variance
MLDisp: Medial-lateral displacements of the COM
MLrange: Medial-lateral range of the COM
PStand: Sit-to-stand power
PTurnSit: Turning and sit power
ST: Single task
Vrange: Vertical range of the COM
